# Evaluation of the Morphology of Ganglion Cell Complex and Functional Outcomes after Internal Limiting Membrane Peeling with Macular Abrasion in Idiopathic Macular Hole

**DOI:** 10.1155/2020/8891057

**Published:** 2020-12-19

**Authors:** Aurelio Imburgia, Purva Date, Alessandro Mularoni, Gian Maria Cavallini, Rodolfo Mastropasqua, Matteo Forlini

**Affiliations:** ^1^Department of Ophthalmology, San Marino State Hospital, Cailungo, San Marino; ^2^Valvekar Medical & Research Centre, Solapur, India; ^3^Institute of Ophthalmology, University of Modena, Modena, Italy

## Abstract

**Aim:**

This study aims to evaluate the morphology of ganglion cell complex (GCC) along with functional outcomes in patients undergoing vitrectomy with ILM peeling and macular abrasion with Tano diamond dusted membrane scrapers (DDMS) for three different stages of the idiopathic macular hole (IMH).

**Methods:**

This retrospective study was conducted between April 2019 and December 2019. 33 patients with IMH were included and divided into three groups: stage I, stage II, and stage IV. All patients were subjected to vitrectomy with ILM peeling. Gentle and vigorous macular abrasion was additionally performed for stage II and stage IV patients, respectively. The best-corrected visual acuity (BCVA), GCC thickness (measured by spectral domain-optical coherence tomography (SD-OCT)), and photopic contrast sensitivity (Rodenstock CV 900 Chart Panel) were determined before surgery and at 1- and 3-month follow-ups.

**Results:**

Closure of MH was achieved in all the patients. The difference between the preoperative and one- and three-month postoperative values of BCVA was statistically significant in the three groups (*P* < 0.01). Contrast sensitivity progressively improved in all patients and was statistically significant (*P* < 0.01). The reduction in GCC thickness during follow-up was 34%–42% of the preoperative measurements. On comparing the mean GCC thickness of the operated and healthy eyes, it was not statistically significant in stage I patients. However, the same when done in stage II and IV was statistically significant with *P* value < 0.05 and *P* < 0.01, respectively.

**Conclusion:**

Combining ILM peeling with macular abrasion in advanced stages of MH may facilitate its closure without significantly affecting the functional outcome.

## 1. Introduction

Idiopathic macular holes are full-thickness defects in the neurosensory retina, which usually results in moderate to severe central vision loss [1, 2]. Pathogenesis of macular holes is due to anomalous vitreomacular traction and incomplete posterior vitreous detachment (IC-PVD). It often leaves remnants of the vitreous cortex on the internal limiting membrane (ILM) surface [3, 4]. ILM is the basal lamina of inner retina. It is formed by the footplates of Muller cells. The structural interface between the retina and the vitreous is composed of collagen fibers, glycosaminoglycans, laminin, and fibronectin.

Kelly and Wendel in the late 80 s performed a pilot study of vitrectomy with ILM peeling as a possible solution to relieve traction over the macula in full thickness macular holes (FTMH). Prior to this, there was no definitive treatment for idiopathic macular holes (IMH) [5].

With the evolution in surgical techniques such as small gauge vitrectomy, epiretinal membrane peeling (ERM), ILM peeling, and inverted flap technique, the percentage of hole closure approached 90–100%, with a low recurrence rate [6–8]. It is proposed that ILM peeling is an adjuvant therapy for inducing controlled gliosis, which helps in hole closure [9]. An inverted flap technique has improved the prognosis of large holes (>500 *μ*m in diameter) from an anatomical point compared with the classic ILM peeling vitrectomy [7]. However, the restoration of the photoreceptor layer (IS/OS junction) and the external limiting membrane (ELM) is not achieved in all patients. Hence, this method may be associated with a poor functional result [10].

ILM peeling itself can lead to visible changes of the inner retinal surface. It may lead to thinning of ganglion cell complex (GCC) [11]. The retinal nerve fiber layer (RNFL) may provide the appearance of “dissociated optic nerve fiber layer” (DONFL) [12, 13]. These changes are linked to a decrease in retinal sensitivity and increase the incidence of microscotomas [12, 14]. Initial studies suggested that DONFL appearance does not affect the retinal function [15, 16]. However, a recent study points toward decreased retinal sensitivity on microperimetry in the area of the DONFL [14].

In recent years, the development of spectral domain-optical coherence tomography (SD-OCT) has allowed detailed study of the retinal layers [17]. The functional potential of the inner retina and recovery of vision is linked to GCC thickness and integrity of the IS/OS junction and ELM [18, 19]. Mahajan et al. [20] proposed macular abrasion technique aiming to eliminate tangential traction. This may aid closure of holes by facilitating approximation of its edges and also allow reconstitution of the IS/OS junction and ELM [21].

The purpose of this study is to evaluate the morphological and functional changes in GCC, in patients undergoing vitrectomy with ILM peeling and macular abrasion with Tano diamond dusted membrane scrapers (DDMS) for IMH.

## 2. Materials and Methods

### 2.1. Study Design

This is a nonrandomized retrospective study carried out on 33 patients (33 eyes) with IMH. All cases were examined and treated between April 2019 and December 2019 in San Marino State Hospital, Istituto per la Sicurezza Sociale, Department of Ophthalmology, Republic of San Marino. All patients underwent 25-gauge vitrectomy (E.V.A D.O.R.C, NE) with ILM peeling using Tano DDMS with or without macular abrasion. All patients were informed about risks and benefits of the surgery, giving written informed consent. The study was conducted in accordance with the tenets of the Helsinki Declaration.

Thirty-three patients (33 eyes) with IMH in different clinical stages were recruited. The MHs were staged by a modified Gass [22] classification, after analyzing fundus images, OCT, medical records, and operative notes.

Consequently, the macular hole patients were classified as stages I, II, and IV. The hole size was calculated on the OCT scans by drawing a horizontal line connecting its two narrowest points, with the line being parallel to the retinal pigmented epithelium. All patients' demographic information was collected from the database. All of them underwent a complete ocular examination before and after surgery, including measurements of best-corrected visual acuity (BCVA), slit-lamp examination, applanation tonometry, fundus examination, photopic contrast sensitivity curve (CS), and SD-OCT evaluation (Spectralis OCT; Heidelberg Engineering GmbH, Heidelberg, Germany).

Pseudophakic patients with IMH with recent onset of diminution of vision (DOV) (less than 3 months) were included. Exclusion criteria were the presence of cataract that represents a bias for the preoperative study of visual acuity and contrast sensitivity, as well as comorbidity affecting visual functions (ERM, diabetic retinopathy, age-related macular degeneration, vascular occlusions, myopic degeneration, inflammatory diseases, trauma, etc.). Follow-up was in the first and third postoperative months.

### 2.2. Surgical Technique

The surgical procedure was a 25-gauge, 3-port pars plana vitrectomy (PPV) performed by experienced surgeons (A.I. and M.F.). A posterior vitreous detachment (PVD) was induced if not already present. A complete vitrectomy was performed. Cases with an evident ERM were excluded to avoid the effect of ERM peeling on retinal tissue. The ILM was stained with Membrane Blue-Dual™ (MBD), consisting of a sterile combination of trypan blue (0.15%), brilliant blue G (0.025%), and 4% polyethylene glycol (PEG). The dye was allowed to stay inside only for the short period required for removing the cannula and inserting the next instrument. After staining, defect was created at the temporal quadrant with the Tano DDMS (Synergetics Inc., O'Fallon, MO, USA) and then peeling flap with Eckardt forceps. The ILM was removed over the entire macular area, with an extension of approximately 2 disc diameters. Before performing the fluid-air exchange, macular abrasion was performed. This entailed soft massage of the edges of the hole within an area of 1 disc diameter with the Tano DDMS, in a radial and centripetal manner (Video 1–link shared in Annexure).

The type of abrasion strokes performed was different as per stage and size of the hole. For stage I, no massage was given. For stage II, gentle strokes were given, whereas for stage IV, relatively vigorous strokes were applied. The aim of macular abrasion was to reduce the size of the hole and facilitate its closure.

Subsequently, the fluid-air exchange and air-gas exchange were performed, using 20% sulfur hexafluoride (SF6). Postoperatively, all patients were instructed to maintain strict prone position for first 3 days and 3-4 hours per day for subsequent 4 days.

During the follow-up, following outcomes were recorded:

SD-OCT was performed to assess anatomical closure of the macular hole. It also allowed evaluation of GCC morphology. GCC thickness was measured in an 8 × 8 mm square centered on the fovea, analyzing the thickness values of the perifoveal quadrants of the three single layers constituting the GCC (RNFL, GCL, and IPL). The reproducibility of the measurement of GCC was confirmed by multiple observations. The preoperative and postoperative GCC thickness map of the affected eye was also compared with that of the contralateral healthy eye, using it as a reference for a normal value of our sample regarding GCC thickness.

Further functional improvement was assessed by noting 2 or more line improvement in BCVA on ETDRS chart. Contrast sensitivity (CS) was evaluated during the follow-up under photopic condition (85 candela/m^2^) using sine-wave gratings, according to Michelson contrast [23] (Rodenstock CV 900 Chart Panel, Germany). The test allows determining the patient's contrast sensitivity curve using circular graphic stimuli containing sinusoidal gratings of different spatial frequencies and different levels of contrast sensitivity. Five spatial frequency levels from A to E, each of this consisting of 8 contrast sensitivity levels, were checked. For each stimulus, the patient must recognize the inclination of the grating, responding with the following 4 possibilities: right, left, vertical, or unrecognized. At the end of the examination, the contrast sensitivity curve of each patient is obtained, indicating the sensitivity value for each spatial frequency. We obtained 5 sensitivity values (between 0 and 8) for the 5 spatial frequency levels from *A* to *E*.

### 2.3. Statistical Analysis

Statistical analysis was performed using Minitab, version 15.1.0.0, statistical software (Minitab Inc., State College, PA, USA). Changes in visual acuity, contrast sensitivity, and GCC thickness were analyzed using the Student paired data test (Student's *t*-test). *P* values less than 0.01 were considered statistically significant.

## 3. Results

**Figure 1:**
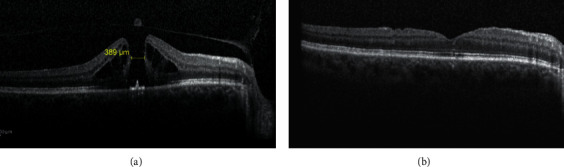
(a) OCT image showing stage II macular hole with a diameter of 389 *μ*m preoperatively. (b) OCT at 3-month follow-up showing closed MH with restored ELM and IS/OS junction integrity.

**Figure 2:**
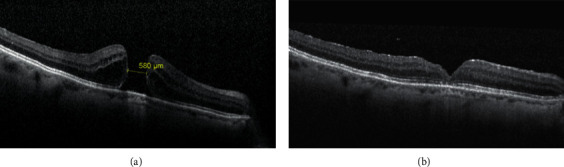
(a) OCT image showing stage IV IMH with a diameter of 580 *μ*m preoperatively. (b) Follow-up OCT scan at 3 months showing closed macular hole with restored ELM and IS/OS junction integrity.

The MH was closed in all cases after the initial surgery (Figures 1 and 2). No intraoperative or postoperative complications were noted. Mean age of patients was 68.57 ± 8.05 years (range 48–81). Twenty patients (60.6%) were females, whereas remaining 13 (39.4%) were males. Seven patients had stage I MH, 9 patients (27.3%) had stage II MH, and remaining 17 patients (51.5%) had stage IV MH. The mean hole diameter was 187.00 ± 65.07 *μ*m for stage I MH, 304.33 ± 81.10 *μ*m for stage II MH, and 533.65 ± 80.26 *μ*m for stage IV MH.  Table 1 shows demographic data and mean preoperative hole diameter values.

**Figure 3:**
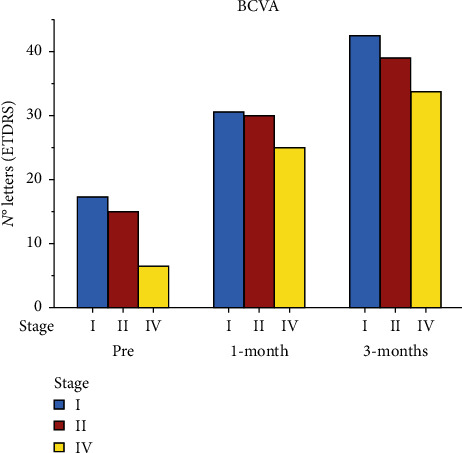
Preoperative and postoperative BCVA.

BCVA improved in all patients after the surgery (Table 2, Figure 3). Preoperative BCVA was 17.14 ± 10.38 letters for stage I MH, 15.11 ± 8.23 letters for stage II MH, and 6.29 ± 3.05 letters for stage IV MH. Postoperative BCVA progressively improved during the follow-up. After 1 month, BCVA improved to 30.71 ± 6.65 letters for stage I MH, 30.11 ± 7.02 letters for II stage MH, and 25.29 ± 8.35 letters for IV stage MH. At third postoperative month, BCVA was 42.57 ± 5.19 letters for stage I MH, 39.22 ± 6.85 letters for II stage MH, and 33.88 ± 8.35 letters for IV stage MH. The difference between the preoperative and one- and three-month postoperative values of BCVA was statistically significant in the three groups (*P* < 0.01).

During the follow-up, the average visual acuity recovery was similar in all stages. Stage I MH patients had an average gain of 26 letters (from 17.14 ± 10.38 to 42.57 ± 5.19) at the final follow-up. Similarly, stage II MH had an average final gain of 24 letters (from 15.11 ± 8.23 to 39.22 ± 6.85). In patients with stage IV MH, VA improved from 6.29 ± 3.05 in the preoperative to 33.88 ± 8.35 letters in the final follow-up with an average final gain of 27 letters at the ETDRS.

Contrast sensitivity (CS) was evaluated in 5 levels, from A to E, each of which has a specific spatial frequency and a different contrast value. Preoperative and postoperative CS are presented in Table 3 for all examined groups. Preoperative CS was *A*: 3.57; *B*: 3.28; *C*: 2.00; *D*: 0.71; and *E*: 0.28 for stage I MH, whereas it was *A*: 1.77; *B*: 1.66; *C*: 0.33; *D*: 0.00; and *E*: 0.00 for stage II MH; and it was *A*: 0.94; *B*: 0.47; *C*: 0.05; *D*: 0.00; and *E*: 0.00 for stage IV MH.

After 1 month, CS was improved, that is, *A*: 4.42; *B*: 4.85; *C*: 3.42; *D*: 1.57; and *E*: 0.85 for stage I MH. For stage II MH, it increased to *A*: 3.88; *B*: 3.22; *C*: 1.66; *D*: 0.77; and *E*: 0.11; and *A*: 3.17; *B*: 3.00; *C*: 1.35; *D*: 0.52; *E*: 0.05 for stage IV MH. At the third postoperative month, CS was *A*: 5.42; *B*: 5.85; *C*: 3.71; *D*: 2.14; and *E*: 1.28 for stage I MH; for II stage MH, it was *A*: 5.00; *B*: 4.66; *C*: 2.77; *D*: 1.33; and *E*: 0.33; and *A*: 4.23; *B*: 4.05; *C*: 2.47; *D*: 1.23; *E*: 0.35 for stage IV MH.

**Figure 4:**
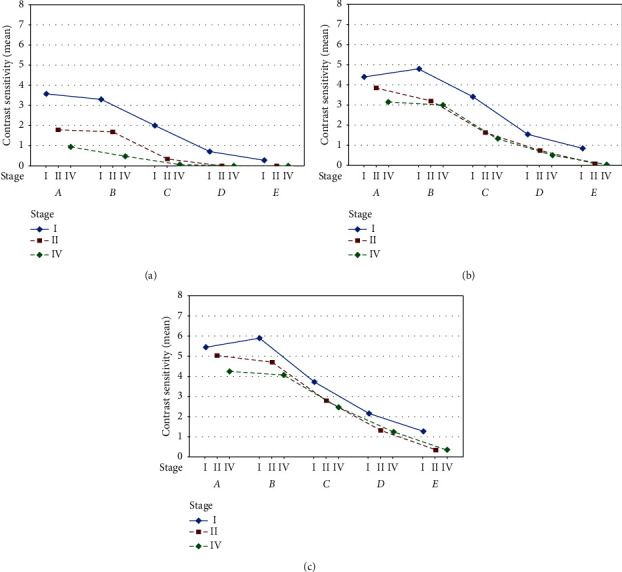
Mean preoperative and postoperative contrast sensitivity (CS) for all groups: (a) preoperative, (b) 1-month follow-up, and (c) 3-month follow-up.

Contrast sensitivity improved during the follow-up in all three groups (Figure 4). The difference between the preoperative and postoperative contrast sensitivity values was statistically significant (*P* < 0.01) in both the first and third month.

**Figure 5:**
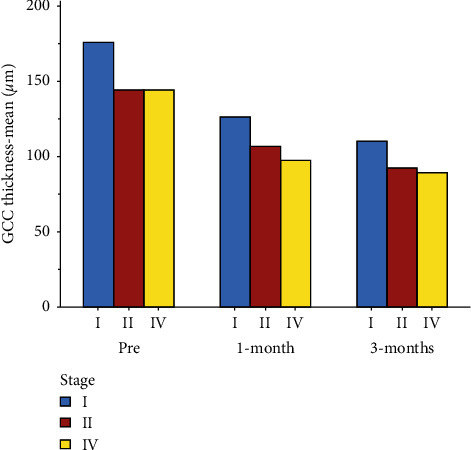
Mean preoperative and postoperative GCC thickness values in the three groups.

The total GCC thickness gradually decreased during the follow-up (Table 4, Figure 5). After the first month, it was 126.25 ± 23.75 *μ*m for stage I MH, 106.50 ± 25.17 *μ*m for stage II MH, and 97.57 ± 14.88 *μ*m for stage IV MH. At the third-month follow-up, the thickness further decreased to 109.96 ± 16.38 *μ*m for stage I MH, 92.94 ± 20.99 *μ*m for stage II MH, and 89.10 ± 14.37 *μ*m for stage IV MH. The difference between the preoperative and postoperative average thickness values was statistically significant (*P* < 0.01) in both the first and third month.

**Figure 6:**
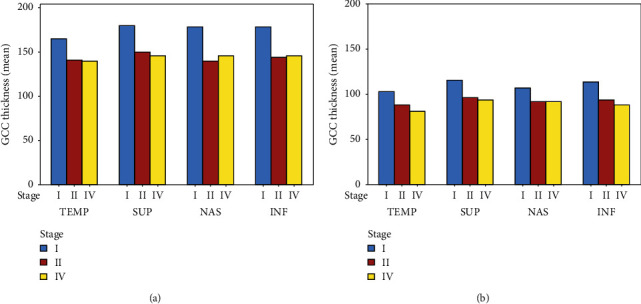
Mean preoperative and postoperative GCC thickness of the perifoveal quadrants of three groups: (a) preoperative GCC thickness of perifoveal quadrants and (b) 3-month GCC thickness of perifoveal quadrants.

**Figure 7:**
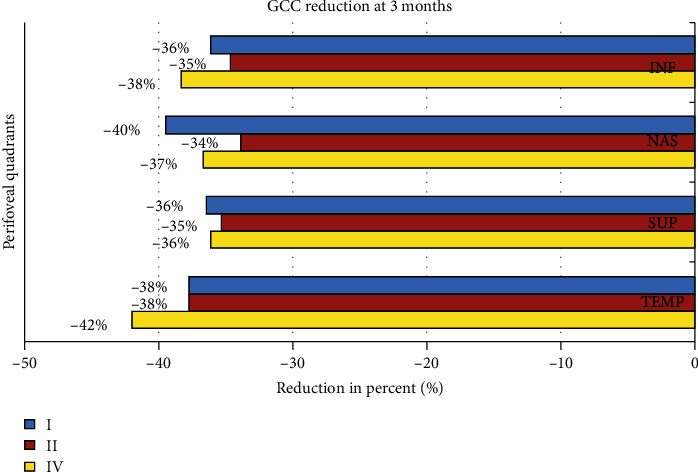
Reduction in thickness of the singular perifoveal quadrants during follow-up.

The analysis of the GCC thickness of the perifoveal quadrants also showed a progressive reduction in thickness in the third month after surgery and was statistically significant (*P* value <0.01) (Table 5, Figure 6). The reduction in thickness of the singular perifoveal quadrants during follow-up was between 34% and 42% of the preoperative values (Figure 7).

**Figure 8:**
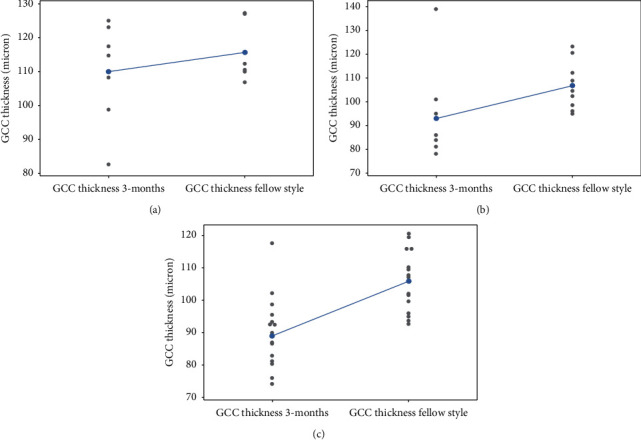
Comparison between GCC thickness of the operated eye and contralateral healthy eye in the three groups at three-month follow up: (a) macular hole stage I, (b) macular hole stage II, and (c) macular hole stage IV.

Finally, mean GCC thickness values of the operated eyes was compared with that of the contralateral healthy eyes, using them as a reference value for our sample (Table 6, Figure 8). Mean total GCC thickness of the fellow healthy eyes was 115.57 ± 8.29 *μ*m for stage I MH, 106.72 ± 10.28 *μ*m for stage II MH, and 105.94 ± 8.83 *μ*m for stage IV MH.

In stage I MH, there was no statistically significant difference between the mean thickness values of the GCC of the operated and healthy eyes, with an average thickness difference between the two groups being 5 *μ*m (*P* > 0.01).

In stage II and IV MH, there was a statistical significant difference between the average GCC thickness of the operated eyes and healthy eyes with *P* value < 0.05 and *P* < 0.01. The average thickness difference between the two groups was 13 *μ*m and 16 *μ*m, respectively.

## 4. Discussion

ILM is the inner most layer of retina constituting the basement membrane and originates from the Muller cells. The outer surface of ILM is continuous with Muller cell end-feet and is adherent to the retinal nerve fiber layer and ganglion cell layer. The etiopathogenesis of MH formation is not clearly understood. Multiple factors such as tangential and anteroposterior traction and degenerative and cellular changes have been speculated. Removal of ILM ensures elimination of residual cortical vitreous, ERMs, and vitreous-derived cells that may be left on the retinal surface [24, 25]. It has been postulated that ILM peeling could activate Muller cells to secrete collagen, basement membrane components, and inflammatory factors. This stimulates glial cell-mediated closure of macular holes. This may explain the modestly higher closure rates observed with ILM peeling compared with vitrectomy alone [26]. In addition, more recent data suggest that MHs may reopen at lower rates when the ILM is peeled [27].

Our results showed that all the IMHs included in the study were closed after 25G PPV with ILM peeling and macular abrasion (wherever indicated). SD-OCT showed restoration of the foveal profile with integrity of the ELM and the photoreceptor layer in all patients. Sabater et al. [28] and Baba et al. [11] also had a 100% MH closure rate on OCT evaluation in 25 and 28 study eyes, respectively.

Visual acuity progressively improved throughout the follow-up in the three groups. All patients had an average gain of more than 4 lines at the ETDRS, with a statistically significant difference between the preoperative and postoperative values (*P* < 0.01).

Contrast sensitivity progressively increased in all stages. The highest mean contrast sensitivity values were found, at the end of the follow-up, in patients with stage I MH, followed by stage II and IV MH. The greatest increase in CS for stage I MH and stage II MH was for levels C, D, and E. For stage IV MH, the greatest gain was for levels A and B.

MBD dye was used to stain the ILM and facilitate peeling. Some dyes, such as indocyanine green, have been associated with retinal toxicity and may be responsible for reducing the GCC. MBD, on the other hand, has been shown to be cytoprotective against retinal nerve cells [29]. Baba et al. [30] showed its influence on GCC reduction to be minimal. Similarly, Sevim and Sanisoglu [31] showed no significant decrease of average superior and inferior GCC thickness after BBG-assisted ILM peeling. Additionally, we ensured bare minimum dye retina contact time by quick aspiration of the dye.

The presence of ganglion cells on the surgically excised ILM, demonstrated by immunohistochemistry, confirms mechanical removal of ganglion cells during peeling, which is suggestive of iatrogenic damage [32]. This damage can be assessed by measuring average GCC thickness after ILM peeling and macular abrasion.

In our study, the mean GCC thickness showed a reduction in all stages during follow-up. The major reduction occurred in the first month after surgery and continued with a low progression till the third month. The sharp reduction in thickness during the first month could be linked to the removal of ILM and resolution of intraretinal edema; the minimum reduction that occurred in the third month may be attributed to iatrogenic damage.

According to some reports, the GCC thickness in normal eyes, as measured by RTVue-100, ranged from 93.7 to 95.1 mm. [33–35] In our study, GCC thickness of unaffected fellow eyes was 108.66 ± 4.6 mm. The postoperative GCC thickness in our study was 96.66 ± 8.8 mm at 3 months, and it was thinner than normal GCC thickness by approximately 11.33 mm.

We compared the mean GCC thickness of the operated eyes with that of the healthy contralateral eyes. In stage I MH, the difference between the two thicknesses was not statistically significant (*P* > 0.05). Thus, it can be concluded that there was not much iatrogenic damage in this group. Stage II MH patients showed statistically significant difference between the two thickness values (*P* value <0.05). In stage IV MH, statistical difference was highly significant (*P* value <0.01).

We also compared the GCC thicknesses of the individual perifoveal quadrants preoperatively and postoperatively. We noticed uniform reduction of the GCC in all 4 quadrants in all stages. The reduction was between 34 and 42% compared with the preoperative values. The higher reduction in thickness at the end of the follow-up was detected in the temporal quadrant. Similar findings were also noted by Baba et al. [30]. Sabater et al. [28] also noted significant reduction of retinal ganglion cell inner plexiform layer (GCIPL) thickness more at the temporal quadrants during analysis. It was done with newer ganglion cell analysis (GCA) software of the Cirrus HD-OCT at 6 months after BBG-assisted ILM peeling vitrectomy.

This could be because of mechanical manipulation of the ILM that always started from the temporal quadrant of the retina. We feel safer to initiate ILM peeling starting from the temporal quadrant because the terminals of retinal nerve fibers exist at the temporal retina. This could have altered the temporal GCC thickness more than that in other quadrants. Contrary to our rationale, Nukada et al. [36] noted similar temporal GCC loss even though initial ILM flap was created at the superior or inferior quadrant.

The nerve fiber layer is physiologically thinner in the temporal quadrant. The density of the ganglion cells in the temporal retina is less than that of its nasal counterpart [37]. These aspects may also be contributing to its iatrogenic damage.

The Tano DDMS is a safer instrument since it only removes the cell membranes and surface layer of the ILM. The abrasion of the MH edge is performed to reduce the size of the large holes and facilitate the reconstitution of IS/OS junction and ELM and possibly stimulate proliferation of glial cells. Michalewska et al. [7] hypothesized that the proliferation of glial cells leads to relocation of adjacent photoreceptors to the fovea, thus explaining the improvement of functional results. However, the improvement in visual acuity and contrast sensitivity confirm the functional success of this technique [21]. Moreover, the use of the Tano DDMS could be beneficial in recurrent patients previously treated with ILM peeling alone.

## 5. Conclusion

To conclude, we studied an alternative method of MH surgery that appears to preserve the retinal function. Although our current practice continues to perform complete ILM peels, the additional macular abrasion technique when selectively applied seems to improve functional outcomes.

The major limitation of this study is the retrospective design with a short observation period and limited subjects. Functional postoperative evaluation could possibly be more accurate with microperimetry and also multifocal electroretinographic evaluations. However, the results are encouraging. Further prospective studies with longer follow-up may be needed to exclude any long-term iatrogenic damage.

## Figures and Tables

**Table 1:** Demographic characteristics and preoperative values.

Parameter	Values	Percentage
*Eye*		
Right	16	48.49
Left	17	51.51

*Macular hole stage*		
I	7	21.2
II	9	27.3
IV	17	51.5

*Hole diameter*		
I	187.14 ± 65.07 *μ*m	
II	304.33 ± 81.10 *μ*m	
IV	533.65 ± 80.26 *μ*m	

**Table 2:** Preoperative and postoperative best-corrected visual acuity.

Best-corrected visual acuity (BCVA)–*N*° letters (ETDRS)			
Stage	Mean *N*°	St Dev	SE mean
*Preoperative values*			
I	17.14	10.38	3.92
II	15.11	8.23	2.74
IV	6.29	3.05	0.74

*1-month postoperative values*			
I	30.71	6.65	2.51
II	30.11	7.02	2.34
IV	25.29	8.35	2.02

*3-month postoperative values*			
I	42.57	5.19	1.96
II	39.22	6.85	2.28
IV	33.88	8.35	2.02

Preoperative and postoperative best-corrected visual acuity (BCVA) *N*° letters (ETDRS). St dev: standard deviation; SE: standard errors.

**Table 3:** Mean preoperative and postoperative contrast sensitivity (CS) for all groups.

CS	Stage I MH	Stage II MH	Stage IV MH
*Preoperative values (mean* *±* *St dev)*			
*A*	3.57 ± 1.51	1.77 ± 0.97	0.94 ± 1.02
*B*	3.28 ± 2.13	1.66 ± 1.41	0.47 ± 0.62
*C*	2.00 ± 2.16	0.33 ± 0.50	0.05 ± 0.24
*D*	0.71 ± 1.25	0.00 ± 0.00	0.00 ± 0.00
*E*	0.28 ± 0.75	0.00 ± 0.00	0.00 ± 0.00

*1-Month postoperative values (mean ± St Dev)*			
*A*	4.42 ± 1.27	3.88 ± 1.26	3.17 ± 1.28
*B*	4.85 ± 1.06	3.22 ± 1.20	3.00 ± 1.36
*C*	3.42 ± 0.97	1.66 ± 0.86	1.35 ± 0.86
*D*	1.57 ± 1.61	0.77 ± 1.09	0.52 ± 0.62
*E*	0.85 ± 1.46	0.11 ± 0.33	0.05 ± 0.24

*3-Month postoperative values (mean ± St Dev)*			
*A*	5.42 ± 0.78	5.00 ± 0.50	4.23 ± 1.20
*B*	5.85 ± 0.69	4.66 ± 1.11	4.05 ± 1.24
*C*	3.71 ± 0.75	2.77 ± 0.83	2.47 ± 0.94
*D*	2.14 ± 0.69	1.33 ± 0.50	1.23 ± 0.75
*E*	1.28 ± 0.95	0.33 ± 0.50	0.35 ± 0.60

**Table 4:** Mean preoperative and postoperative GCC thickness values in the three groups.

Ganglion cell complex thickness (*μ*m)			
Stage	Mean	St Dev	SE mean
*Preoperative values*			
I	175.85	32.18	6.08
II	144.08	36.84	6.14
IV	144.58	31.91	3.87

*1-Month postoperative values*			
I	126.25	23.75	4.48
II	106.50	25.17	4.19
IV	97.57	14.88	1.80

3-Month postoperative values			
I	109.96	16.38	3.09
II	92.94	20.99	3.49
IV	89.10	14.37	1.74

**Table 5:** Mean preoperative and postoperative GCC thickness of the perifoveal quadrants of three groups.

Quadrant	Stage I MH	Stage II MH	Stage IV MH
*Preoperative values (mean* *±* *St Dev)*			
Temporal	165.57 ± 24.95	141.78 ± 33.60	140.52 ± 35.93
Superior	180.14 ± 41.44	149.22 ± 45.35	146.18 ± 30.98
Nasal	178.86 ± 34.29	140.22 ± 35.60	146.17 ± 35.13
Inferior	178.86 ± 31.28	145.11 ± 37.83	145.52 ± 27.51

*3-Month postoperative values (mean* *±* *St Dev)*			
Temporal	103.28 ± 16.21	88.11 ± 13.95	81.29 ± 9.89
Superior	114.42 ± 13.80	96.44 ± 20.42	93.17 ± 14.71
Nasal	108.00 ± 15.05	92.55 ± 28.56	92.41 ± 17.77
Inferior	114.14 ± 20.67	94.66 ± 21.36	89.52 ± 11.88

**Table 6:** Comparison between GCC thickness of the operated eye and contralateral healthy eye in the three groups at three-month follow-up.

*GCC thickness of stage I MH at 3-month follow-up*					
	Mean	St Dev		SE mean	
Operative eye	109.96	14.04		5.68	
Fellow eye	115.57	8.29		3.13	

*Estimation for paired difference*					
Mean	St Dev	SE mean	95% CI for *μ*_d_	*T* value	*P* value
−5.607 *μ*m	9.500	3.591	(−14.393; 3.179)	−1.56	<0.1694

*GCC thickness of stage II MH at 3-month follow-up*					
	Mean	St Dev		SE mean	
Operative eye	92.94	18.99		6.33	
Fellow eye	106.72	10.28		3.42	

*Estimation for paired difference*					
Mean	St Dev	SE mean	95% CI for *μ*_d_	*T* value	*P* value
−13.778 *μ*m	13.689	4.563	(−24.300; −3.255)	−3.02	<0.0166

*GCC thickness of stage IV MH at 3-month follow-up*					
	Mean	St Dev		SE mean	
Operative eye	89.10	11.10		2.69	
Fellow eye	105.94	8.83		2.142	

*Estimation for paired difference*					
Mean	St Dev	SE mean	99% CI for *μ*_d_	*T* value	*P* value
−16.838 *μ*m	10.214	2.477	(−24.074; −9.603)	−6.80	<0.0001

St Dev: standard deviation; SE mean: standard error of mean; CI: confidence interval.

## Data Availability

The data are available on request.

## Supplementary Materials

Supplementary Materials Video 1 showing vitrectomy with MBD assisted ILM peeling with macular abrasion for stage IV MH. Video attached. .
